# Atypische Neuritis nervi optici (NNO): Bedeutung einer umfassenden Abklärung

**DOI:** 10.1007/s00347-020-01165-8

**Published:** 2020-07-23

**Authors:** R. Yaïci, A. Carzoli, F. Bétis, G. Geerling, R. Guthoff, T. Guthoff

**Affiliations:** 1 Klinik für Augenheilkunde, Universitätsklinikum Düsseldorf, Moorenstr. 5, 40225 Düsseldorf, Deutschland; 2 service d’ophtalmologie, CHPG, 1 avenue Princesse Grace, 98000 Monaco, Monaco

**Keywords:** Neuromyelitis optica, NMSOD, Anti-MOG-Antikörper, Bilaterales Papillenödem, Postvakzinal, Neuromyelitis optica, NMSOD, Anti-MOG antibodies, Bilateral papilledema, Postvaccination

## Abstract

Eine 65-jährige Frau wurde wegen plötzlicher beidseitiger Sehminderung überwiesen, nachdem sie kurz zuvor geimpft wurde. Augenärztlich zeigte sich beidseits eine ausgeprägte Papillenschwellung. In der Magnetresonanztomografie (MRT) fand sich keine zerebrale Beteiligung oder transverse Myelitis. Serologisch konnten wir Myelin-Oligodendrozyten-Glykoprotein(MOG)-IgG nachweisen, sodass wir mit Hochdosiskortikosteroidpulstherapie behandelten. *Diskussion*: Bei atypischer Optikusneuritis muss an eine Neuromyelitis-optica-Spektrum-Erkrankung (NMOSD) gedacht werden, die durch die Bestimmung von Aquaporin 4(AQP4)- und MOG-IgG weiter charakterisiert werden sollte.

## Abstract (en)

A 65-year-old woman was referred for sudden bilateral loss of vision. She was vaccinated a few days earlier. The ophthalmological examination showed a massive optic disc swelling on both eyes. Magnetic resonance imaging (MRI) revealed suspected bilateral optic neuritis without cerebral involvement or transverse myelitis. After serological detection of anti-MOG (myelin oligodendrocyte glycoprotein) antibodies, the patient was treated with high-dose corticosteroid pulse therapy until vision recovered. *Discussion*: an atypical optic neuritis may indicate a neuromyelitis optica spectrum disorder (NMOSD), which should be further characterized by determination of Aquaporin 4(AQP4)-IgG and MOG-IgG.

## Falldarstellung

### Anamnese

Eine 65-jährige Frau wurde von ihrem niedergelassenen Augenarzt wegen einer bilateralen Papillenschwellung und plötzlicher beidseitiger Sehminderung innerhalb von wenigen Stunden überwiesen. Zusätzlich klagte die Patientin über Übelkeit und Erbrechen. Kopfschmerzen wurden von der Patientin verneint. Die Anamnese ergab außer einem moderaten Nikotinabusus keine Auffälligkeiten. Einige Tage zuvor hatte die Patientin eine Impfung mit Revaxis®, Sanofi (Paris, Frankreich), gegen Diphtherie, Tetanus und Poliomyelitis erhalten.

### Befund

**Figure 1:**
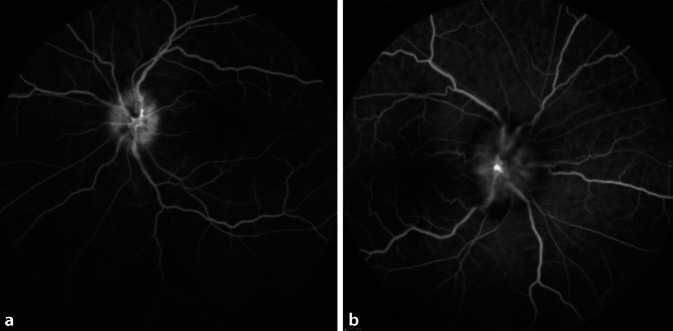

Der Visus war am rechten Auge auf 0,1, am linken auf Fingerzählen reduziert. Der Ishihara-Test zeigte eine bilaterale Rot-Grün-Schwäche und einen relativen afferenten Pupillendefekt links. Am Fundus zeigte sich beidseits eine ausgeprägte Papillenschwellung mit peripapillären flammenförmigen Hämorrhagien ohne Zeichen einer Vaskulitis, Vitritis oder Netzhaut‑/Aderhautbeteiligung. In der Fluoreszeinangiographie (FAG) stellten sich Papillenleckagen (Abb. 1) dar. Eine kranielle und spinale MRT ergab den Verdacht auf eine bilaterale Sehnervenentzündung ohne Hinweis auf eine transverse Myelitis, zerebrale Beteiligung oder Raumforderung. Eine Biopsie der A. temporalis superficialis, eine Lumbalpunktion mit Liquordruckmessung und eine kardiovaskuläre Abklärung waren unauffällig. Labordiagnostisch zeigten sich keine erhöhten Entzündungs- oder Infektionsparameter (Borrelien, Lues, Bartonellen, Toxoplasmose) Auch Anti-Neutrophile cytoplasmatische Antikörper (ANCA), antinukleärer Antikörper (ANA), nativer Anti-DNA-Ab und extrahierbare nukleäre Antigene(ENA) waren negativ. Die Bestimmung von Aquaporin 4(AQP4)- und Myelin-Oligodendrozyten-Glykoprotein(MOG)-Antikörpern ergab schließlich den Nachweis von MOG-IgG.

### Diagnose

Wir stellten die Diagnose einer *Anti-MOG-Antikörper-vermittelten Neuritis nervi optici, einer Neuromyelitis-optica-Spektrum-Erkrankung (NMOSD)*.

### Therapie und Verlauf

**Figure 2:**
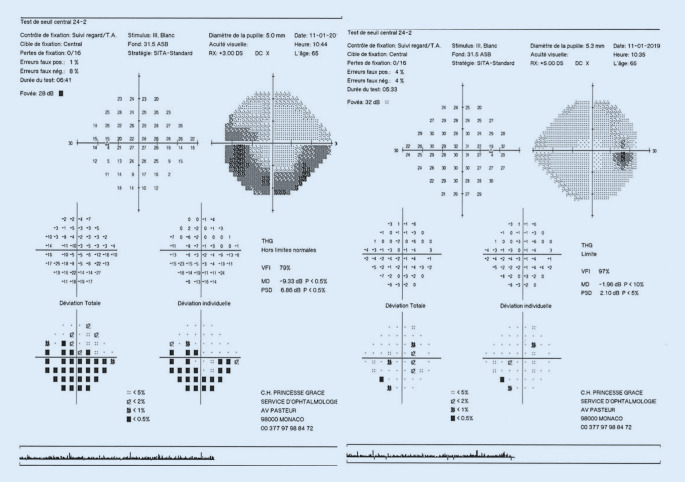

Eine hoch dosierte intravenöse Methylprednisolon-Therapie 1 g/Tag für 5 Tage und anschließend 1 mg/kgKG über 4 Wochen ausschleichend reduziert, führte zu einer Visuserholung auf rechts 0,8 und links 1,0 und einem Rückgang der Papillenschwellung innerhalb von 4 Wochen. Die 30°-Perimetrie war 4 Wochen nach Therapie rechts nahezu regelrecht, links bestand noch ein bogenförmiges Skotom unten mit einem vergrößerten blinden Fleck (Abb. 2).

## Diskussion

In unserem Fall handelte es sich nach den durch das *International Panel for NMO Diagnosis (IPND)* neu definierten Kriterien von 2015 [1] um eine Neuromyelitis-optica-Spektrum-Erkrankung (engl. „neuromyelitis optica spectrum disorders“ [NMOSD]). Bis 2006 wurde diese Erkrankung auch als Neuromyelitis optica (NMO) oder historisch als Devic-Krankheit bezeichnet [2].

Kardinalsymptom dieses Krankheitskomplexes auf ophthalmologischem Fachgebiet ist die *atypische Neuritis nervi optici *[2, 3].

Die typische Neuritis nervi optici, wie sie bei der multiplen Sklerose (MS) vorkommt, zeigt eine einseitige Visusminderung mit Bulbusbewegungsschmerz, relativem afferentem pupillärem Defizit, Farbentsättigung und Zentralskotom bei Frauen in jungem Alter [4]. Von einer atypischen Neuritis nervi optici spricht man beim Vorliegenden folgender Kriterien [4]:

- Alter unter 18 oder über 50 Jahre,
- fehlender Augenbewegungsschmerz,
- simultan beidseitiges Auftreten,
- Papillitis mit starker Schwellung, Randblutungen, harten Exsudaten, Cotton-wool-Spots oder initialer Optikusatrophie.

Erkrankungsmechanismen, die zu einer atypischen Sehnerventzündung führen können und die mittels MRT und Serologie differenziert werden müssen, sind neben der NMOSD Autoimmunerkrankungen (z. B. Sarkoidose, Lupus erythematodes oder „chronic relapsing inflammatory optic neuropathy“ [CRION]) sowie ein infektiöses (z. B. Borreliose, Lues oder Bartonellen), postinfektiöses oder postvakzinales Geschehen [4].

Der atypischen NNO im Rahmen von NMOSD gehen häufig grippeähnliche Prodromi voraus. Typischerweise kommt es dann zu einem schnellen beidseitigen Visusverlust, wobei das zweite Auge meist wenige Stunden oder Tage nach dem ersten betroffen ist, was bis zur Erblindung führen kann. Die Papillenschwellung kann mild oder stark ausgeprägt sein. Typisch ist ein schubförmiger Verlauf (90 %), selten verläuft die Erkrankung monophasisch. Chronisch progrediente Verläufe kommen im Gegensatz zur MS nicht vor [5].

Bei der NMOSD handelt es sich um eine Gruppe seltener chronisch entzündlicher Erkrankungen des zentralen Nervensystems (ZNS). Sie betrifft bevorzugt Frauen (9:1) im mittleren Alter (Altersgipfel 4. Dekade), wobei Erstmanifestationen vom Kindes- bis ins hohe Erwachsenenalter beschrieben sind [5]. Neben der NNO können eine Myelitis, ein Area-postrema-Syndrom und die klinische Beteiligung von Hirnstamm, Dienzephalon oder Großhirn einzeln oder in Kombination auftreten [2]. Eine Triggerung der Demyelinisierung durch Impfungen wie bei unserer Patientin ist in einigen Fallberichten beschrieben [3].

Ein wesentliches Diagnosekriterium der NMOSD ist der Serumantikörpernachweis von AQP4-IgG oder MOG-IgG, deren Vorhandensein die Erkrankung klar von der MS abgrenzt. AQP4 ist ein Wasserkanalprotein, das beim Menschen in Astrozyten vorkommt [6], es kommt also primär zu einem Astrozytenschaden [2]. AQP4-IgG sind bei NNO als Erstmanifestation einer NMOSD in 70 % nachweisbar. In ca. 25 % der AQP4-negativen Fälle kann man Antikörper gegen MOG nachweisen [5].

MOG ist ein Bestandteil der Myelinscheiden des ZNS. Es spielt wahrscheinlich eine Rolle bei der Adhäsion der Myelinfasern, der Modulation der Interaktion zwischen Myelin und dem Immunsystem und der Stabilität der Oligodendrozyten [2]. Beide Antikörper zusammen kommen nur in Ausnahmefällen beim gleichen Patienten vor.

Das Vorhandensein der unterschiedlichen Antikörper ist klinisch und prognostisch relevant. Es wird kontrovers diskutiert, ob es sich möglicherweise sogar um unterschiedliche Krankheitsentitäten handelt [2]. Die Patienten mit MOG-IgG vermittelter Erkrankung sind meist etwas jünger (Erkrankungsgipfel 3. Dekade), das Geschlechtsverhältnis ist 1:1. Bei ca. 50 % der Patienten ist eine Neuritis nervi optici erstes Symptom der Erkrankung und tritt auch im weiteren Verlauf häufig auf. In 50 % ist diese beidseitig und meist mit einer Papillenschwellung verbunden [7].

Während bei AQP4-positiven Patienten die einzelne Neuritisepisode einen stärkeren Sehnervenschaden hinterlässt, scheint es bei MOG-IgG-positiven Patienten zwar zu einer schnelleren Visuserholung, aber zu häufigeren Rezidiven zu kommen, was zu einem vergleichbaren Nervenfaserverlust führt [8].

Die Akuttherapie der NMOSD besteht in Methylprednisolon i.v. 1 g/Tag für 3 bis 5 Tage mit mindestens 4‑wöchiger Ausschleichphase [2, 9, 10], sofern keine absoluten Kontraindikationen vorliegen. Bei ausbleibender Besserung kann eine Plasmapherese oder Immunadsorption zum Einsatz kommen [2].

Bei AQP4-IgG-positiver NMOSD ist die unvollständige Remission der Schübe oft Ursache dauerhafter Behinderung, daher ist eine langfristige Immuntherapie zur Schubprävention in den meisten Fällen indiziert. Analog dazu wird auch bei MOG-IgG-positiver NNO mit Azathioprin, Rituximab oder Mycophenolat-Mofetil behandelt, wenn auch eine Langzeittherapie wahrscheinlich nicht bei allen Patienten erforderlich ist und individuell entschieden werden muss. Spezifische Studien, betreffend den Effekt von Rituximab oder neuerer Medikamente wie Interleukin-6-Inhibitoren (Tocilizumab), Komplementsysteminhibitoren (Eculizumab) oder dem seit Juni 2020 von der Federal Drug Administration für AQP4-positive Patienten zugelassenen CD19-Antikörper Inebilizumab-cdon, fehlen bei der Subgruppe der MOG-IgG-positiven Patienten bislang [11].

Einige häufige Immuntherapeutika der MS wie Fingolimod, Interferon‑β und Natalizumab können eine NMOSD teils massiv verschlechtern [3], auch deshalb ist die Unterscheidung zwischen beiden Erkrankungen wichtig.

Der Augenarzt spielt in der Erkennung der atypischen Neuritis nervi optici eine wichtige Rolle und kann durch eine zielführende Anamnese und Diagnostik die Weichen für die weitere Abklärung stellen.

## Fazit für die Praxis

- Eine Differenzierung zwischen typischer und atypischer Neuritis nervi optici ist für die weitere Therapie essenziell.
- Bei atypischer NNO ist ein wichtiger Teil der Diagnostik die Bestimmung von AQP4-IgG und MOG-IgG.
- Die Evaluation und Therapie der NMOSD muss interdisziplinär erfolgen.
